# Multiplex polymerase chain reaction as an improved method for screening *Histoplasma capsulatum* mating types

**DOI:** 10.1590/0074-02760180340

**Published:** 2018-09-17

**Authors:** Fernando Almeida-Silva, Leonardo Silva Barbedo, Maria Lucia Taylor, Mauro de Medeiros Muniz, Allan Jefferson Guimarães, Rosely Maria Zancopé-Oliveira

**Affiliations:** 1 Fundação Oswaldo Cruz-Fiocruz, Instituto Nacional de Infectologia Evandro Chagas, Laboratório de Micologia, Setor de Imunodiagnóstico, Rio de Janeiro, RJ, Brasil; 2 Universidade Federal do Amazonas, Instituto de Saúde e Biotecnologia, Coari, AM, Brasil; 3 Universidad Nacional Autónoma de México, Facultad de Medicina, Departamento de Microbiología-Parasitología, DF, México; 4 Universidade Federal Fluminense, Instituto Biomédico, Departamento de Microbiologia e Parasitologia, Niterói, RJ, Brasil

**Keywords:** mating type, MAT1, Histoplasma capsulatum, multiplex PCR

## Abstract

Histoplasmosis is a systemic mycosis infection caused by *Histoplasma capsulatum*, a heterothallic ascomycete. The sexual reproduction of this fungus is regulated by the mating type (*MAT1*) locus that contains *MAT1-1* and *MAT1-2* idiomorphs, which were identified by uniplex polymerase chain reaction (PCR). This study aimed to optimise single-step multiplex PCR for the accurate detection of the distinct mating types of *H. capsulatum.* Among the 26 isolates tested, 20 had *MAT1-1* genotype, while six showed *MAT1-2* genotype, in agreement with the uniplex PCR results. These results suggest that multiplex PCR is a fast and specific tool for screening *H. capsulatum* mating types.

The significance of sexual selection, the component of natural selection associated with variation in mating success, is well established for the evolution of animals and plants but not fungi. Mating types determine genetic compatibility among fungal gametes and are important for sexual selection in two respects. First, genes at the mating-type loci regulate different aspects of mating and thus may be subject to sexual selection. Second, for sexual selection, not only the two sexes (or sex roles) but also the mating types can form classes, wherein the members compete for access to members of the other class.^1^ In ascomycetes, the genes of the two mating types are not homologous and are therefore referred to as idiomorphs.^2^ Depending on the species, the genes at the mating-type loci may regulate functions that operate during mating such as extracellular signalling,^3^ ^,^ ^4^ cell fusion,^5^ inheritance of cytoplasmic genes,^6^ and establishment of a diploid or heterokaryotic individual.^7^ In addition, these genes may regulate cell division,^8^ sexual reproduction,^9^ and virulence^10^ after zygote formation.

Mating loci have been identified in other filamentous ascomycete fungi, including *Neurospora crassa*,^11^ ^,^ ^12^ *Aspergillus nidulans*,^13^ and *A. fumigatus*.^14^ Mating process has a potential role in the virulence of human pathogens. Recombination between two strains may result in a new strain with increased virulence.^15^ The pathogenic fungus *Histoplasma capsulatum* has been shown to undergo recombination;^16^ ^,^ ^17^ however, little is known about its mating process on molecular levels.

*H. capsulatum* is a heterothallic ascomycete that has anamorph and teleomorph stages. The anamorph stage or asexual reproduction occurs upon hyphal fragmentation or conidium production in a specialised hypha or by budding in yeasts. The sexual or teleomorph stage occurs by the conjugation of two compatibility types, encoded by the locus *MAT1*, and contains two idiomorphic regions as follows: *MAT1-1* (alpha-domain transcription factor) and *MAT1-2* [high-mobility-group (HMG) transcription factor] also known as - and +, respectively.^10^ ^,^ ^18^ It has been suggested that strains of each mating type are not equally represented among clinical isolates of *H. capsulatum*, with a current ratio of 7:1 (*MAT1*-*1*:*MAT1-2*); however, environmental samples exhibit a 1:1 ratio of the two mating types.^19^ Experiments with murine infection revealed that the two mating types showed no difference in their virulence potential.^20^ A recent study using African clinical isolates showed a ratio of 1:3 (*MAT1*-*1*:*MAT1-2*), contradicting other results.^21^

Molecular methods are routinely applied for the identification of mating types in fungi since phenotypic methods are unpredictable; fungi such as *H. capsulatum* lose the ability to mate *in vitro* within a short period of time.^22^ Among the molecular methods, uniplex polymerase chain reaction (PCR) using specific primers targeting idiomorphic regions *MAT1-1* and *MAT1-2* is the most common technique.^15^ A recent study using this technique compared the frequency of the locus *MAT1* in Mexican and Brazilian isolates and found mating types in 14 Mexican isolates (four *MAT1-1* and 10 *MAT1-2*) and 14 Brazilian isolates (all were *MAT1-1*).^23^

Until now, uniplex PCR has been applied to detect mating types in *H. capsulatum*.^18^ Here, we evaluate a multiplex PCR format for the ability to differentiate between *MAT1-1* and *MAT1-2* in *H. capsulatum* strains and compare the results with those of uniplex PCR using the same primer sets (Table I).

We used the sequences of the two reference strains from GenBank, G217B: *MAT1-1* (accession number EF433757, ATCC 26032) and G186A: *MAT1-2* (accession number EF433756, ATCC 26029) to perform an *in silico* multiplex PCR assay using the FastPCR v.6.0 software with two sets of primers (Table I). We observed that the primers specific for the control reference strains generated fragments of 440 and 528 bp, as previously described.^15^

To confirm our *in silico* observations, genomic DNA was extracted from the yeast phase of reference strains,^24^ and multiplex PCR was performed with the primers previously described (Table I) in a final volume of 50 µL. Each reaction mixture contained 75 ng of DNA, 1X PCR buffer [10 mM Tris-HCl (pH 8.4) and 50 mM potassium chloride (KCl)], 1.5 mM magnesium chloride (MgCl_2_) (Thermo Scientific TM, Brasil), 200 µM of each dNTP (Thermo Scientific TM, USA), 2.5 U platinum DNA Taq polymerase (Thermo Scientific TM, Brasil), and 50 ng of each primer. Multiplex PCR was performed on a Bio-Rad C1000 Thermal Cycler with the following program: Initial denaturation at 95ºC for 3 min, followed by 35 cycles of denaturation for 30 s at 95ºC, annealing for 30 s at 58ºC, extension for 1 min and 30 s at 72ºC, and a final extension step at 72ºC for 10 min.^23^ The samples were analysed by electrophoresis on a 1% agarose gel at 100 V for approximately 1 h. The gel was stained with ethidium bromide (0.5 μg/mL) and the amplicons were observed and photographed under UV light (280 nm).

Multiplex PCR reproducibility was confirmed by repeating the assays at least thrice under same conditions. Three different conditions evaluated *in silico* to standardise this protocol were as follows: (i) reference strain G186A DNA idiomorph (*MAT1-2*); (ii) a DNA mix of both reference strains (*MAT1-2 + MAT1-1)*; and (iii) reference strain G217B DNA idiomorph (*MAT1-1*) (Fig. 1).

The results of multiplex PCR showed DNA fragments of 440 and 528 bp for *MAT1-1* and *MAT1-2*, respectively. A clear resolution of bands obtained for the mixed samples allowed distinction between the two idiomorphic regions. This enabled us to perform classification in both the reference samples and the sample sets studied (Fig. 2).

Uniplex and multiplex PCR were performed following the above protocol for 26 Brazilian *H. capsulatum* isolates from the Fungal Culture Collection of Evandro Chagas National Institute of Infectious Diseases, Oswaldo Cruz Foundation (INI/Fiocruz) (Table II). Of these isolates, 14 were previously characterised as *MAT1-1* by uniplex PCR^23^ and were re-evaluated by uniplex and multiplex PCR. All of these isolates, previously determined as *MAT1-1* idiomorph by uniplex PCR,^23^ displayed the same genotype by both methods. However, analysis of the 26 Brazilian isolates showed that *MAT1-1* genotype was predominant and represented 77% (20) of the isolates, whereas 23% (6) of the isolates were of *MAT1-2* genotype. Overall, there was 100% agreement between uniplex and multiplex PCR results for mating type characterisation (Table II).

Here, we demonstrated a significant improvement in the molecular determination of mating types of *H. capsulatum* by comparing the results between the developed protocol and the previously known methodology.^15^ Multiplex PCR assay showed accurate and specific results, as evident from the similarity with uniplex PCR results.^23^ Several reports are focused on the development of simple and low-cost methodologies as well optimisation of existing molecular methods. New approaches or modifications have already been proposed. For instance, PCR in multiplex format for laboratory diagnosis of *Pneumocystis jirovecii*, *H. capsulatum*, and *Cryptococcus neoformans*/*C. gattii* ^25^ and detection of *C. parapsilosis* complex species has been reported.^26^

**TABLE I:** Primers for the multiplex polymerase chain reaction^(15)^

Primers	Sequence
*MAT1-1* S (Forward)	5’-CGTGGTTAGTTACGGAGGCA-3’
*MAT1-1* AS (Reverse)	5’-TGAGGATGCGAGTGATGGGA-3’
*MAT1-2* S (Forward)	5’-ACACAGTAGCCCAACCTCTC-3’
*MAT1-2* AS (Reverse)	5’-TCGACAATCCCATCCAATACCG-3’

**Fig. 1:**
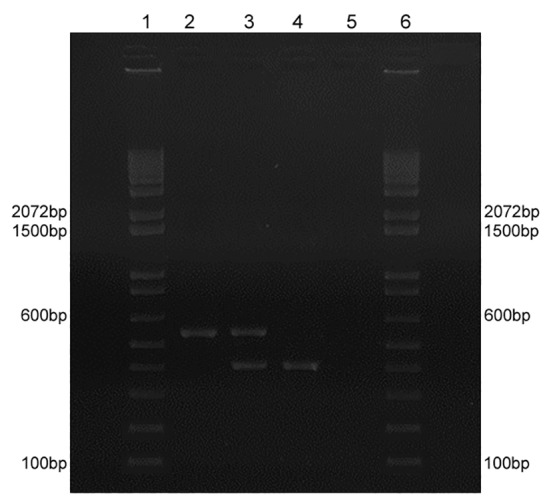
representative multiplex polymerase chain reaction profiles of *Histoplasma capsulatum* mating types. (1 and 6) Molecular marker 100-bp DNA ladder (Thermo Scientific); (2) reference strain G186A (ATCC 26029) *MAT1-2* corresponding to 528 bp; (3) reference strains G186A (higher fragment with 528 bp) and G217B (lower fragment with 440 bp); (4) reference strain G217B (ATCC 26032) *MAT1-2* corresponding to 440 bp; (5) negative control.

**Fig. 2:**
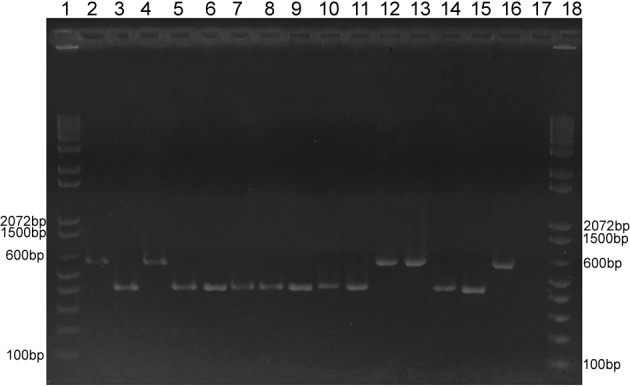
representative gel electrophoresis of multiplex polymerase chain reaction profiles from 13 studied isolates and reference strains. Lanes 1 and 18 = 100-bp DNA ladder (Thermo Scientific); lanes 2, 4, 12, and 13 corresponding to isolates 128H, HC40039, INI 01/16, and IPEC 01/12, respectively, classified as mating type 1-2; lanes 3, 5, 6, 7, 8, 9, 10, 11, and 14 corresponding to the isolates 39439, INI 02/16, 18H, 37307, M396/08, M487/08, IGS19, RPS51, and IPEC 02/13, respectively, classified as mating type 1-1; lane 15 = reference strain G217B mating type 1-1 (ATCC 26032); lane 16 = reference strain G186A mating type 1-2 (ATCC 26029); lane 17 = negative control.

**TABLE II:** Mating types of 26 Brazilian *Histoplasma capsulatum* isolates

Isolate	Source	Location	Mating type	
			Uniplex PCR	Multiplex PCR
18H	Human	RJ	*MAT1-1*	*MAT1-1*
37307	Human	RJ	*MAT1-1*	*MAT1-1*
247BL	Human	MS	*MAT1-1*	*MAT1-1*
M396/08	Animal	SP	*MAT 1-1*	*MAT 1-1*
M1084/08	Animal	SP	*MAT1-1*	*MAT1-1*
M487/08	Animal	SP	*MAT1-1*	*MAT1-1*
M975/08	Animal	SP	*MAT1-1*	*MAT1-1*
AC05	Soil	RJ	*MAT1-1*	*MAT1-1*
TI01	Soil	RJ	*MAT1-1*	*MAT1-1*
IGS19	Soil	RJ	*MAT1-1*	*MAT1-1*
RPS51	Soil	RJ	*MAT1-1*	*MAT1-1*
CO2	Soil	RJ	*MAT1-1*	*MAT1-1*
CO4	Soil	RJ	*MAT1-1*	*MAT1-1*
IGS4/5	Soil	RJ	*MAT1-1*	*MAT1-1*
129H	Human	RJ	*MAT1-2*	*MAT1-2*
39942	Human	RJ	*MAT1-2*	*MAT1-2*
HC40039	Human	RJ	*MAT1-2*	*MAT1-2*
INI 01/16	Human	RJ	*MAT1-2*	*MAT1-2*
INI 06/16	Human	RJ	*MAT1-2*	*MAT1-2*
IPEC 01/12	Human	RJ	*MAT1-2*	*MAT1-2*
2090603	Human	RJ	*MAT1-1*	*MAT1-1*
39439	Human	RJ	*MAT1-1*	*MAT1-1*
HC 18	Human	RJ	*MAT1-1*	*MAT1-1*
INI 02/16	Human	RJ	*MAT1-1*	*MAT1-1*
INI 03/16	Human	RJ	*MAT1-1*	*MAT1-1*
IPEC 02/13	Human	RJ	*MAT1-1*	*MAT1-1*

*MAT1-1*: mating type 1-1; *MAT1-2*: mating type 1-2; MS: Mato Grosso do Sul; PCR: polymerase chain reaction; RJ: Rio de Janeiro; SP: São Paulo.

This work describes the assessment of mating types of *H. capsulatum* by genomic DNA amplification with a single PCR reaction. The multiplex PCR profiles were highly informative, generated clearly distinct banding patterns for each *MAT1* idiomorph, and allowed easy differentiation. This proposed technique is simple, reliable, rapid, cheap, and requires less technical effort.

In conclusion, multiplex PCR may be performed for the accurate identification of *H. capsulatum* mating types in a simple one-step reaction. There are two remarkable points demonstrated in this work. First, this identification process may be completed in a multiplex PCR step followed by electrophoresis. Second, the method saves time, reagents, and cost. We suggest that the introduction of this method in research laboratories for mating type identification may be relevant for epidemiological surveillance and virulence studies. This method may be of real importance for gathering relevant information in *H. capsulatum* culture databank.
